# Chitosan degradation products promote healing of burn wounds of rat skin

**DOI:** 10.3389/fbioe.2022.1002437

**Published:** 2022-10-11

**Authors:** Chuwei Zhang, Qingrong Zhang, Dongmei Yang, Yating Qiao, Bolin Wang, Jun Yan, Zihan Li, Zhanghao Huang, Youlang Zhou, Kesu Hu, Yi Zhang

**Affiliations:** ^1^ Department of Burn and Plastic Surgery, Affiliated Hospital of Nantong University, Medical School of Nantong University, Nantong, China; ^2^ Department of Burn and Plastic Surgery, Affiliated Hospital of Nantong University, Nantong, China; ^3^ Third Military Medical University (Army Medical University), Chongqing, China; ^4^ Outpatient Treatment Center, Department of Burn and Plastic Surgery, Affiliated Hospital of Nantong University, Nantong, China; ^5^ Department of Gastrointestinal Surgery, Affiliated Hospital of Hebei University, Baoding, China; ^6^ Department of Thoracic Surgery, Affiliated Hospital of Nantong University, Medical School of Nantong University, Nantong, China; ^7^ The Hand Surgery Research Center, Department of Hand Surgery, Affiliated Hospital of Nantong University, Nantong, China

**Keywords:** chitooligosaccharides (COS), deep burn wounds, transcriptome analysis, wound healing, signaling pathways

## Abstract

Burns can impair the barrier function of the skin, and small burns can also cause high mortality. The WHO has described that over 180,000 people die of burns worldwide each year. Thus, the treatment of burn wounds is a major clinical challenge. Chitooligosaccharides (COS) are alkaline amino oligosaccharides with small molecular weights obtained by enzyme or chemical degradation of chitosan. With the characteristics of biocompatibility, water solubility and degradability, it has attracted increasing attention in the fields of biomedicine. In the present study, we used COS to treat deep second-degree burn wounds of rat skin and found that COS was able to promote wound healing. We also revealed that COS could promote fibroblast proliferation. Transcriptome sequencing analysis was performed on COS-treated fibroblasts to identify the underlying mechanisms. The results showed that COS was able to promote wound healing through regulation of the mitogen-activated protein kinase (MAPK) pathway and growth factor Hepatocyte Growth Factor (HGF). Our results provide a potential drug for burn wound therapy and the related molecular mechanism.

## Introduction

Burns are common skin lesions that often impair normal skin function (Mayet et al., 2014). Burn injury is directly related to the extent of the burns (Bayir et al., 2019). In 1995, in cooperation with the World Health Organization (WHO), the International Burn Society published burn treatment guidelines, which described four classifications (superficial, superficial partial thickness, deep partial thickness, and full thickness) of burns (Cope, 1943; Jackson, 1970; Latarjet, 1995). Tissue repair and wound healing are highly regulated physiological processes, including bidirectional interactions of many basic cell types with their cell extracellular matrix and products by which the wound tissue heals themselves (Piraino and Selimović, 2015; Kaparekar et al., 2020). The depth of burn wounds and the occurrence of the infection determine the healing process, time, and morbidity associated with the injury (Whitney and Wickline, 2003; Monstrey et al., 2008; Gurfinkel et al., 2010).

Clinically, superficial partial-thickness burns are usually healed by nonsurgical wound care, while full thickness burns obviously require excision and transplantation to close the wound (Karim et al., 2020). The term “indeterminate-depth burn” (IDB) is often used to describe deeper partial-thickness burns with unknown healing potential, and it represents a complex management challenge for clinicians. The wound healing process is an intricate biological process containing a series of stages: homeostasis, inflammation, proliferation and remodeling, which are affected by the immunostimulatory activities of materials used for wound healing such as growth factors, proinflammatory cytokines, and inflammatory cells (Rowan et al., 2015; Wang et al., 2018). Therefore, the requirement for biomaterials with excellent biological activities such as anti-inflammatory, antibacterial, and promoting healing activities, is increasing rapidly (Salamone et al., 2016; Jafari et al., 2020).

Chitin is the most abundant natural polysaccharide, and chitosan is its most important derivative (Liaqat and Eltem, 2018). Chitosan exhibits a variety of valuable characteristics such as nonpoisonous, biocompatible (Sharma et al., 2018) and noncarcinogenic (Alves and Mano, 2008) properties. Therefore, chitosan has great application potential in the field of biomedicine (Kumar et al., 2016; Kumar et al., 2020). However, owing to its short shelf life and high molecular weight, the water insolubilities of chitosan limit its wide application (Liaqat and Eltem, 2018; Kumar et al., 2020).

Chitooligosaccharides (COS) are water-soluble low-molecular-weight derivatives obtained by the chemical, physical or enzymatic depolymerization of chitin and chitosan (Aam et al., 2010; Liaqat and Eltem, 2018) and are superior to the parent polymers in many aspects (Liaqat and Eltem, 2018). Recently, COS has been proven to have a variety of promising biological activities and has attracted widespread attention in drug therapy (Eaton et al., 2008). In this study, we found that COS can not only promote deep burn wound healing *in vivo* but also promote the proliferation of fibroblasts *in vitro*. We performed transcriptome analysis and Gene Ontology (GO) and Kyoto Encyclopedia of Genes and Genomes (KEGG) annotation analyses on fibroblasts treated with COS and verified the accuracy of the sequencing results.

## Materials and methods

### Preparation of chitooligosaccharides

The preparation of COS was the same as that described previously (Wang et al., 2005; Yang et al., 2009; Wang et al., 2016; Zhao et al., 2017). Briefly, chitosan was added to 15% H_2_O_2_ and the mixture was irradiated for 4 min at 700 W with microwave power. The crude COS was collected by filtration and redissolved in a minimum volume of distilled water, and then the crude COS was purified using a Sephadex-25 column (size of column: 1.6 cm × 85 cm bed volume) (Zhao et al., 2017). COS powder was obtained by lyophilizing the eluted COS solution under vacuum. Endgroup analysis was used to determine the average molecular weight (MW) of COS (Yang et al., 2006). The average degree of polymerization of the purified COS was ∼7 with MW of ∼900 Da. The degree of deacetylation was approximately 92.3% (Zhao et al., 2017).

### Deep second-degree scald model and treatment groups

The animal studies were approved by the Nantong University Animal Experiment Ethics Committee. Healthy male Sprague-Dawley (SD) rats weighing approximately 200 ± 20 g were offered by Nantong University Laboratory Animal Center. The animals were allowed to eat and drink freely and were fed adaptively for 7 days. The back hair of the rats was shaved in advance. One day later, the rats were anaesthetized with 3% sodium pentobarbital (30 mg/kg), and after anesthesia, the scald model was prepared by a temperature control instrument (YLS-5Q; China) (Zhang et al., 2022). Deep burn wounds were formed by placing a 2 cm^2^ instrument probe (temperature set at 85°C) on the back of the rat for 15 s. The rats were sacrificed 48 h after injury, and the depth of skin injury was evaluated by hematoxylin eosin (H&E) staining. The scald model was proven to be deep second-degree burns.

All rats were randomly divided into three groups and were shaved 1 day in advance.

Sham scald group: Eight rats, and no treatment was given after shaving.

Scald control group: Twenty-four rats were scalded on the back and injected with 0.2 ml of physiological saline around the wound subcutaneously every other day for a week.

Scald COS treatment group: Twenty-four rats were scalded on the back and injected with 0.2 ml (0.2 mg/ml) of COS solution around the wound subcutaneously every other day for a week.

The wounds were all exposed, and on the second day and first 6 weeks after injury, pictures of the wounds were taken.

### Morphology of the wounds and measurement of the wound area

Two days and in the first 6 weeks after injury, the rats were fixed, and wound pictures were taken. Then, the wound shape was drawn on transparent paper, and the wound area was determined by the paper weight. The percentage of wound healing rate and the wound complete epithelialization rate was equal to (wound burn mass on initial−wound mass on the specific day)/(wound burn mass on initial) × 100%, as described previously (Zhang et al., 2022).

### Blood perfusion in burn wounds

The wound perfusion of scald rats was measured in the first 3 weeks after injury. After anesthetizing and fixing the rats, a blood perfusion measuring instrument (Laser Doppler Flowmetry, United States) was used to measure the wound blood flow. Then, LDPI image analysis software (LDPIwin, 3.1.3) was used to analyze the wound blood perfusion value (Zhang et al., 2022).

### Tissue water content

Eight rats in each group were randomly selected on the second day and the first 2 weeks after injury. After euthanizing the rats, carefully collected the skin tissue (including tissues within 0.5 cm of the outer edge of the wound) was carefully collected, and the tissue was dried for 48 h. The tissues were weighed before and after drying to calculate the weight difference. The water content = (wet weight−dry weight)/wet weight × 100%, as described previously (Zhang et al., 2022).

### Histopathology analysis

Eight rats were sacrificed randomly in each group in the first 4 weeks after injury, and the wound tissue was collected and placed in 10% formalin solution. The samples were dehydrated, embedded in 10% formalin solution overnight, sliced into sections (4 µm) and stained with H&E. A microscope (Leica DMR 3000; Leica Microsystems, Bensheim, Germany) was used to observe the changes in the skin appendages (sebaceous glands and hair follicles) and the growth of new epithelium.

### Primary fibroblast culture and immunofluorescence analysis

Young SD rats were sacrificed, disinfected with 75% alcohol, and washed with sterile phosphate-buffered saline (PBS) supplemented with 100 μg/ml penicillin/streptomycin three times. The subcutaneous fat, blood vessels, and other tissue of the skin on the backs of SD rats were carefully removed, and the tissue was rinsed several times until it turned white. Then, the skin was cut into 1 mm^2^ pieces. For the isolation of skin fibroblasts, the tissue was preincubated with dispase (2 mg/ml; Sigma) at 37°C for 4 h to remove the epidermis and prevent the contamination of keratinocytes. Then the epidermal layers were removed, and the remaining dermal tissue was added to PBS containing type I collagenase (1 mg/ml, Gibco) and digested in a water bath for 30 min at 37°C. Dulbecco’s Modified Eagle’s Medium (DMEM, high glucose, Gibco) supplemented with 10% fetal bovine serum (FBS, Gibco) and 50 μg/ml penicillin/streptomycin (culture medium) was added to terminate digestion, and the digested cells were then passed through a 70-μm MACS SmartStrainer (Miltenyi Biotec, Germany). The filtered solution was centrifuged for 10 min at 1,000 rpm, the cell precipitate was collected, the culture medium was added, and the cell suspension was centrifuged to wash the cells again. The cells were gently precipitated and cultured in an incubator for 24 h at 5% CO_2_ and 37°C. When the cell density in the culture dish reached 80%–90%, the cells were passaged.

Third-generation fibroblasts were selected for immunofluorescence identification. The cells were inoculated into a 24-well plate with sterile small round slides (3 × 10^4^ cells per well). When the cell confluence reached approximately 70%, the cells were fixed with 4% paraformaldehyde for 30 min, covered with blocking solution, and incubated for 1 h at 37°C. Approximately 200 μl of rabbit anti-vimentin primary antibody (1:200, Proteintech company, #10366-1-AP) was added, and the cells were incubated overnight at 4°C. The cells were covered with the anti-rabbit secondary antibody (1:5000, ABclonal) and incubated for 1 h in the dark at 37°C. The cell nuclei were stained with DAPI (4′,6-diamidino-2-phenylindole, 1:10000, Solarbio, #C0060) for 10 min at room temperature. The cells were washed with PBS each time the solution was changed. A small amount of glycerin mounting tablets was dropped on a clean glass slide, a small round glass slide was retrieved from the 24-well plate, and the round glass slide was placed cell surface side down on the glass slide. Cell morphology was observed with a fluorescence microscope (Leica DMR 3000; Leica Microsystem, Bensheim, Germany), and images were recorded (Yang et al., 2022).

### Cell mitochondrial activity assay

To determine the effects of COS on the mitochondrial activity of fibroblasts, MTT [3-(4, 5-dimethylthiazol-2-yl)-2, 5-diphenyltetrazolium bromide] assays were performed. Fibroblasts were seeded (200 μl, 1 × 10^4^ cells/ml) into 96-well plates and incubated with various concentrations of COS (0, 0.5, 1 and 2 mg/ml) at 37°C, and 200 µl medium was added to each well. Each group had six duplicate wells. After stimulation for 24, 48, and 72 h, 20 µl of MTT (5 mg/ml, Sigma) was added to each well. The cells were incubated for 4 h at 37°C, the medium was removed, and 150 µl of dimethyl sulfoxide (DMSO) was added. After gently shaking the plates in the dark for 10 min, the absorbance D (570) value of the cells was determined at 570 nm on a microplate reader (ThermoFisher, United States). Untreated cells with 100% mitochondrial activity were used as a control, and assays were repeated three times per group (Yang et al., 2018).

### Fibroblast proliferation assay

The fibroblasts were seeded onto 0.01% poly-L-lysine-coated 96-well plates with an initial density of 1 × 10^5^ cells/well in 0.2 ml of culture medium and incubated at 37°C and 5% CO_2_ for 24 h (Yang et al., 2018; Jin et al., 2022). Then, the cells were exposed to COS at concentrations of 0, 0.5 mg/ml and 1 mg/ml for 24 h. Finally, the cells were fixed with 4% formaldehyde for 30 min in PBS. According to the manufacturer’s protocol, a Cell Light EdU DNA Cell Proliferation Kit (Ribobio) was used to assay the fibroblasts after labeling. Under a fluorescence microscope, the proliferation of fibroblasts (ratio of EdU + to all fibroblasts) was analyzed with randomly selected field images. All experiments had three replicates.

### Fibroblast cycle analysis

After seeding cells into 6-well plates (3 ml/well, 1 × 10^6^ cells/ml), they were treated with COS at 0, 0.5 mg/ml and 1 mg/ml for 72 h. The cells were harvested and resuspended in 50 µl PBS. Then, the cell suspension was fixed with ice-cold 70% alcohol and reserved overnight at −20°C. After washing the cells, they were stained for 30 min with a propidium iodide (PI) solution (1 mg/ml RNase A and 0.05 mg/ml PI; Sigma). Finally, AttuneTM NxT software (v2.7.0) was used to test the stained cells on a flow cytometer (Attune NxT, Invitrogen) (Yang et al., 2021).

### Migration assay

To study the effect of COS on the migratory capacity of fibroblasts, a wound healing assay was performed. The fibroblasts (3 ml/well, 1 × 10^6^ cells/ml) were seeded into 6-well plates and incubated at 5% CO_2_ and 37°C. After the cells reached 80%–90% confluence, they were subjected to starvation for 24 h in low serum (1% FBS) medium. The monolayer was scratched with a sterile 1,000 μl pipette tip, and the exfoliated cells were removed with PBS (Xu et al., 2018). Then, the cells were cultured in complete medium supplemented without or with COS (0, 0.5, and 1 mg/ml). At 0, 6, 24, and 48 h postwounding, scratched fields were photographed, and wound closure area was calculated as follows: Migration area = (wound area on initial−the remaining area of wound at each time point)/(wound area on initial) × 100% (Zhu et al., 2022).

The transwell assay was performed using the cell culture insert (FALCON, NJ, United States). Complete medium supplemented without or with COS (0, 0.5 and 1 mg/ml) was added to the 24-well plate as the lower chamber. The cell culture insert (FALCON, NJ, United States) was placed into each well, and the top was pressed lightly to ensure tight bonding (Zhao et al., 2017). Fibroblasts (4 × 10^4^ cells/well) were suspended in low serum medium and seeded into the cell culture insert (FALCON, NJ, United States) with an 8 μm pore size as the upper chamber. After culturing for 24 h, the cells attached to the upper surface of the filter membranes were removed, and the migrated cells on the lower surface were stained for 10 min with 0.5% crystal violet. The migration level was observed under a microscope.

### RNA sequencing and bioinformatics analysis

Total RNA extracted from fibroblasts (treated with 0 or 1.0 mg/ml COS for 24, 48, and 72 h) was subjected to library construction and RNA Sequencing (RNA-seq) analysis after selection by RNA Purification Beads (Illumina, San Diego, CA, United States). Briefly, mRNA was purified from the total RNA of fibroblasts with poly-T oligo-attached magnetic beads (Illumina, San Diego, CA, United States). Then, the RNA was used as a template to synthesize the first strand of cDNA, and the first strand of cDNA was used as a template to synthesize the second strand of cDNA. The remaining protruding ends were converted into flat ends by exonuclease/polymerase activities followed by enzyme removal, and six RNA sequence libraries were constructed. Library preparation and sequencing were performed on an Illumina NovaSeq platform by Personal Biotechnology Cp., Ltd. (Shanghai, China). Raw data has been submitted to the Sequence Read Archive (SRA) database at the National Center for Biotechnology Information (NCBI) with the accession ID PRJNA860830, and Gene function was annotated based on Gene Ontology (GO) and Kyoto Encyclopedia of Genes and Genomes (KEGG). The criteria for differentially expressed genes (DEGs) need to be greater than or less than a twofold change from the control.

### Quantitative real-time PCR analysis

To prove the accuracy of the sequencing results, Hepatocyte Growth Factors (HGFs) were selected to verify whether their trends were consistent with the sequencing results. The cells were cultured for 24, 48, and 72 h with or without 1.0 mg/ml COS, and the cells were collected with TRIzol Reagent (Invitrogen) to extract total RNA to determine HGF expression. cDNA was synthesized from 1 μg of total RNA in a 20 µl reaction system using a reverse transcription kit (TaKaRa) and was detected by a real-time fluorescence quantitative PCR system. The primers were designed and synthesized by RuiMian Biotechnology, and GAPDH was used as an endogenous control. The primer sequences used for PCR were as follows: HGF Forward: 5′-TCT​TGA​CCC​TGA​CAC​CCC​T-3′, Reverse: 5′ -GGT​ATT​GGT​GGT​TCC​CCT​G-3′, GAPDH Forward: 5′ -ATG​GCC​TTC​CGT​GTC​CC-3′, Reverse: 5′-ACC​ACC​TGG​TGC​TCA​GTG​TAG-3′. The PCR conditions were predenaturation at 95°C. There were 45 cycles of denaturation at 95°C for 5 s and annealing at 60°C for 30 s. The Δ cycle threshold (Ct) method was adopted, and the relative expression level was calculated using the comparative 2^−ΔΔ^ method. This experiment was replicated three times.

### Western blot assay

Western blotting was used to detect the expression of some signaling pathway-related proteins in cells (Lai et al., 2015; Hou et al., 2019; Liu et al., 2019; Cao et al., 2021). Fibroblasts were exposed to 1 mg/ml COS for 0, 0.5, 1, 2, 4, 6, 8, and 12 h, and then the fibroblasts were treated with lysis buffer containing protease inhibitors (Yang et al., 2018) and phosphatase inhibitors (Roche, Mannheim, Germany). The supernatant was collected by centrifuging the lysate at 4°C for 20 min at 12,000 RPM. The protein concentrations of different treatment groups were determined with Bio-Rad Protein Assay Reagent (Vancouver, Canada) and adjusted to the same level. The protein solution was mixed with SDS-PAGE sample loading buffer (5×; Biosharp) and denatured at 100°C. The lysate was electrophoretically separated on SDS-polyacrylamide gels and transferred to a polyvinylidene fluoride (PVDF) membrane. Following transfer, TBST buffer (50 mm Tris-HCl, 100 mm NaCl, and 0.1% Tween-20; pH 7.6) was used to wash the membranes, and the membranes were blocked for 2 h with 5% skimmed dry milk/TBST and incubated at 4°C to block primary antibodies. The primary antibodies were as follows: anti-phosphorylated ERK1/2 (p-ERK1/2, #4370, 1:2000, Cell Signaling Technology), anti-ERK (#4695, 1:1000, Cell Signaling Technology), anti-phosphorylated-p38 (#8690, 1:1000, Cell Signaling Technology), anti-p38 (#4511:1000, Cell Signaling Technology), anti-p-JNK (#9255, 1:2000, Cell Signaling Technology), JNK (#9252, 1:1000, Cell Signaling Technology), anti-PCNA (#60097-1-Ig, 1:10000, Proteintech), and anti-GAPDH antibodies (#60004-1-Ig, 1:20000, Proteintech). The following day, the membranes were washed with TBST three times at room temperature and then incubated with a secondary antibody at 4°C overnight. The membranes were washed as mentioned above, and then the Odyssey infrared imaging system (LI-COR, Lincoln, NE, United States) was used to visualize the protein bands (Zhang et al., 2022). Finally, a computer imaging system was used to quantify the optical density of each band and the relative difference between bands on the membrane, and the protein band density was normalized to that of GAPDH.

### Statistical analysis

The results were all expressed as the means ± standard deviations (SD). SPSS statistical software was used to analyze the data, Student’s t test was used for the differences between two groups, and one-way analysis of variance (ANOVA) was used for the differences among three groups. *p* values < 0.05 were considered statistically significant.

## Results

### Establishment of deep second-degree burn models

**FIGURE 1:**
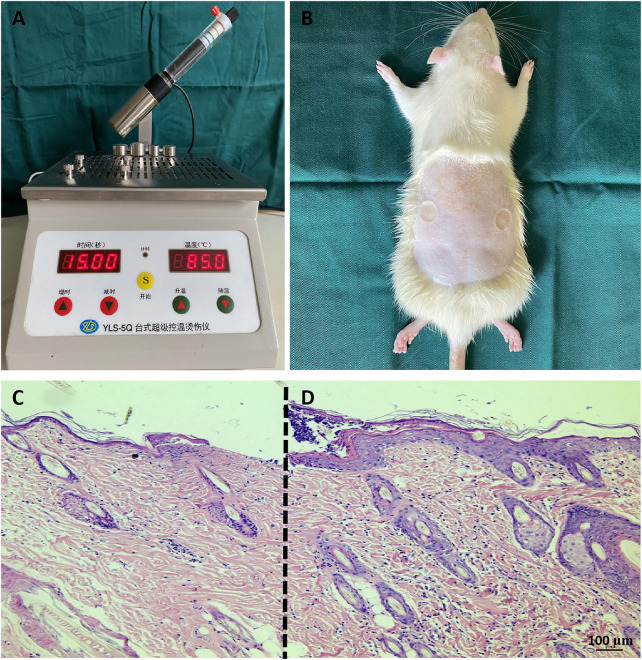
Rat deep second-degree scald models **(A,B)** Establishment of deep second-degree burns. **(C,D)** H&E staining of tissue sections. Compared with the normal skin tissue at the edge of the burn wound **(D)**, the surface of the wound **(C)** was thinner, the tissue was edematous, the cells were obviously shrunken, and the sweat glands and hair follicles were damaged. The scale bar represents 100 µm.

Two regions on the rat back were scalded to form a deep second-degree burn model (Figures 1A,B). The cells were atrophied, the skin appendages were damaged, the subcutaneous tissue was obviously edematous, and the epidermis of the burned skin tissue was significantly thinner (Figure 1C) compared with the normal skin around the wound (Figure 1D). The rat deep second-degree burn model was successfully generated.

### Analysis of histopathology following rat burn wounds

On the second day and the first 6 weeks after injury, the rats were fixed and pictures of the wound were taken with a camera. The wounds of rats were almost completely healed 3 weeks after injury, and the new epithelium was pink and thin.

**FIGURE 2:**
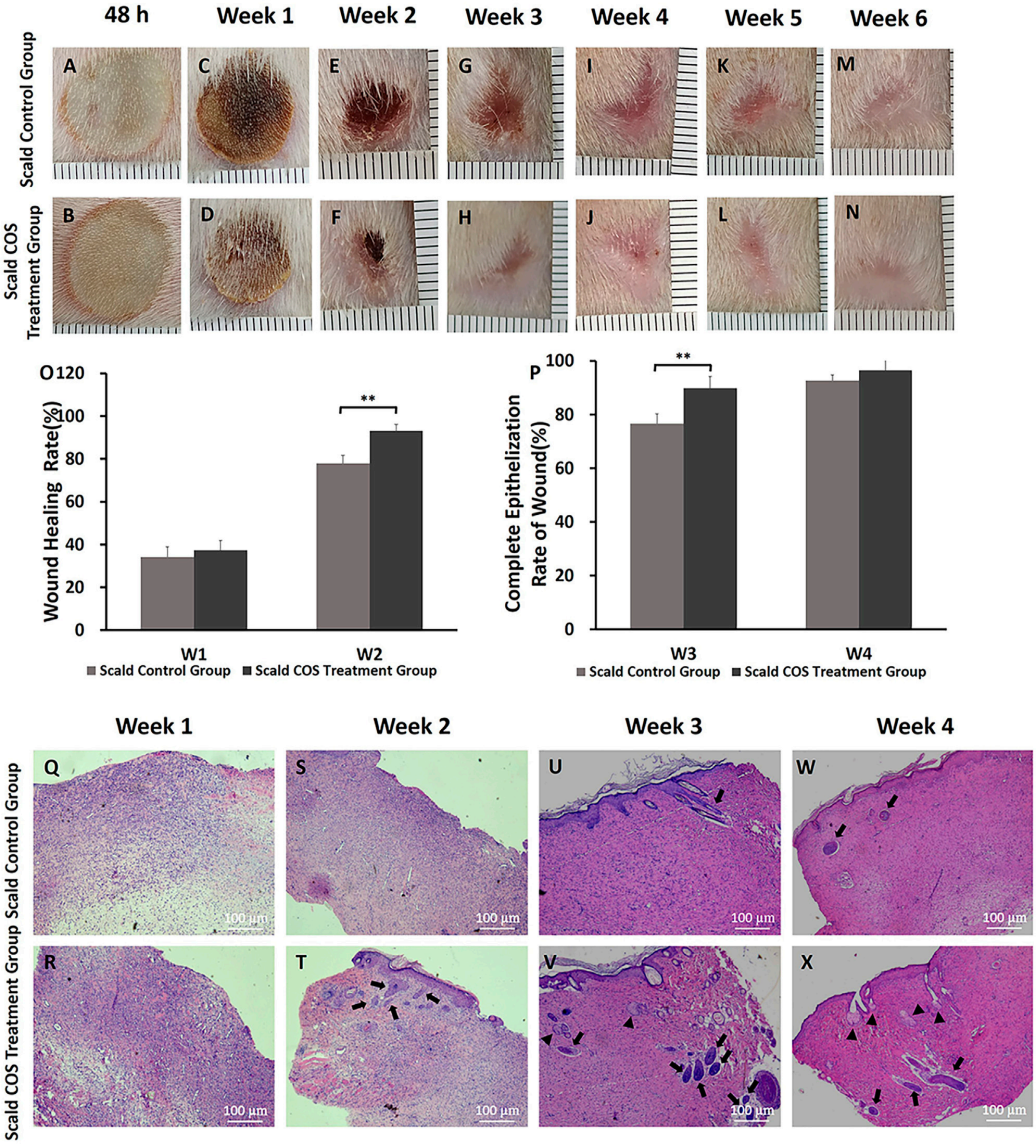
Wound healing, wound healing rate, complete epithelialization rate of burn wounds, and histopathology analysis in rats **(A–N)** Typical wound images of rats in the two scald groups within 6 weeks after injury. **(O)** Wound healing rate in the first week and the second week after injury **(P)** Complete epithelialization rate on the third week and the fourth week after injury. **p < 0.01. **(Q–X)** H&E staining of wounds of the two scald groups on the first 4 weeks after injury. The number of newborn sebaceous glands and hair follicles in the COS-treated group was more than that in the scald control group. Triangles point sebaceous glands, and thick arrows point newborn hair follicles. The scale bar represents 100 µm.

At the early stage after injury, wound edema was obvious. Then, scabs formed on the wound surface (Figures 2A,B). One week after injury, the scab around the wound gradually fell off (Figures 2C,D), and there was no significant difference in the wound healing rate at the first week after injury between the two scald groups (*p* > 0.05) (Figure 2O). At the second week after injury, some scabs began to fall off, new epithelium formed on the edge of the wounds (Figures 2E,F), and the wound healing rate of the COS treatment group was higher (*p* < 0.01) (Figure 2O). On the third week after injury, wounds in both groups almost healed completely, but the new epithelium was pink and thin (Figures 2G,H), and the rate of complete epithelialization of the wound in the COS treatment group was significantly higher (*p* < 0.01) (Figure 2P). Subsequently, the color of the skin faded gradually (Figures 2I–N), and at the fourth week after injury, there was no significant difference in the wound complete epithelialization rate between the two groups (*p* > 0.05) (Figure 2P).

To determine the changes in skin and skin appendages in burn wounds, a microscope was used to observe the wound sections after H&E staining. On the first week after injury, the wounds of rats were scabbed (Figures 2C,D). Microscopically, the wound epidermis was absent, the nuclei were shrunken, necrotic and degenerated, the collagen fibers were degenerated and necrotic, and there were numerous inflammatory cells infiltrated (Figures 2Q,R). The wounds in both groups were significantly contracted 2 weeks after injury (Figures 2E,F). The new collagen fibers in the two groups were disorderly arranged and the collagen connection was loose. (Figures 2S,T). We also found that there were epithelial cells and a small number of skin appendages in the COS group (Figure 2T, thick arrows). On the third week after injury, the wounds of the rats all healed completely (Figures 2G,H). The epithelial cells were well differentiated, and the skin tissue was fully epithelialized. Under the microscope, there were more newborn sebaceous glands (triangles) and hair follicles (thick arrows) in the COS treatment group than in the control group (Figures 2U–X).

### Wound blood perfusion and tissue water content

**FIGURE 3:**
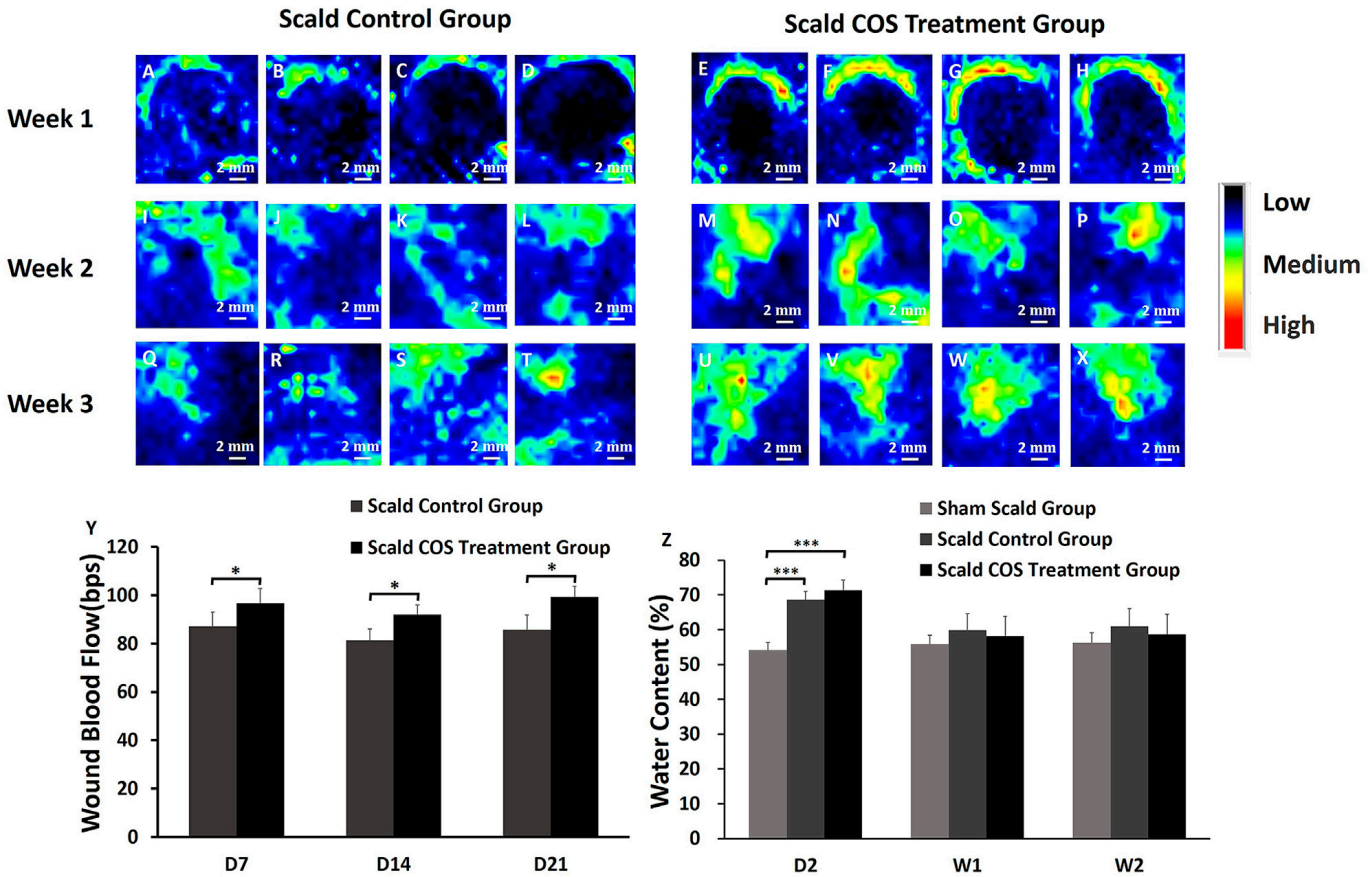
Blood perfusion on the wound surface of rats in the scald groups. Black indicates the area with low or no blood perfusion, and the blood perfusion gradually increases for blue, yellow, green, and red **(A–X)** Typical images of blood perfusion in the wounds of rats. **(Y)** Comparison of blood perfusion on the wounds of the two groups of rats. **(Z)** Comparison of the water content of skin tissue among the three groups. *p < 0.05, ***p < 0.001. The scale bar represents 2 mm.

Rat wound blood perfusion was measured and analyzed. One week after injury, there were high blood flow areas around the edge of the wound, the blood flow around the wound in both groups was higher than that in the wound (Figures 3A–H), and the blood flow of the rats of the control group was significantly lower than that of the COS treatment group (*p* < 0.05) (Figure 3Y). Two weeks after injury, the wound edge blood flow in both groups was decreased, and the blood flow in the control group was still significantly lower than that of the COS treatment group (*p* < 0.05) (Figures 3I–P,Y). The low wound blood flow both disappeared in both groups, which means that the rats had healed completely. Three weeks after injury, the blood flow of the wound in the control group was still significantly lower than that of the COS treatment group (*p* < 0.05) (Figures 3Q–Y).

The skin tissue of the three groups of rats was collected, and the water content of the tissue was analyzed. The tissue water contents of the two scald groups was significantly higher on the second day after injury than those of the sham scald group (Figure 3Z; *p* < 0.001), indicating that after injury, the wound tissue edema was obviously edematous. The water contents of the sham scald group were slightly lower than those of the two scald groups 1 week after injury, while there was no significant difference (*p* > 0.05).

### Isolation and primary culture of rat skin fibroblasts

**FIGURE 4:**
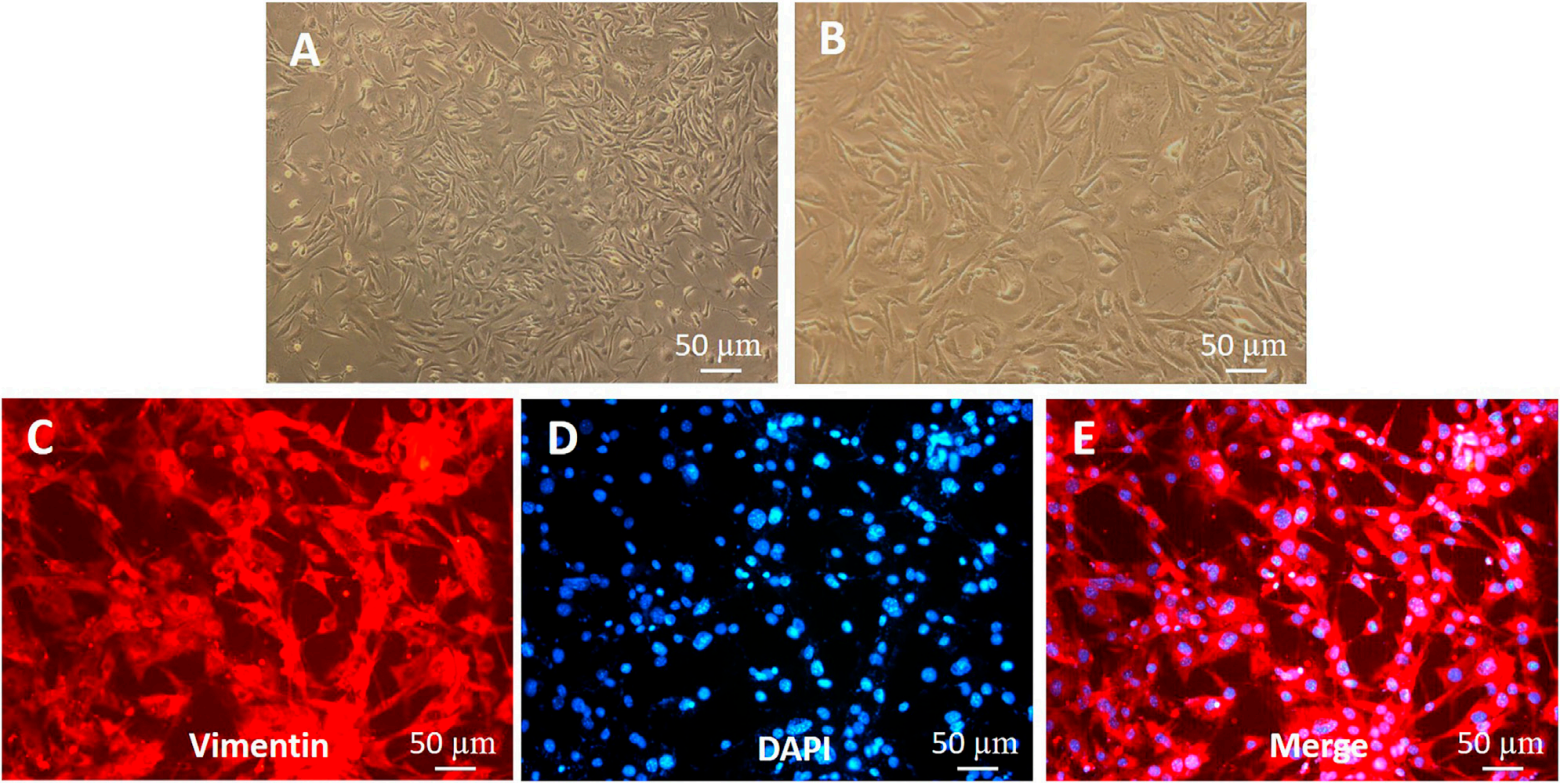
Identification of fibroblasts **(A,B)** Morphology of fibroblasts. **(C–E)** Vimentin expression in fibroblasts (immunofluorescence staining). The shorter scale bar represents 50 μm, the middle scale bar represents 100 μm, and the longer scale bar represents 50 µm.

The primary fibroblasts were cultured by enzyme digestion with the back skin of young SD rats. In the process of culture, the number of fibroblasts increased gradually, the cytoplasm of cells extended pseudopodia, the cells were long spindle or irregular polygon shaped, and the cells grew in whirlpool and radial shape (Figures 4A,B). Vimentin is a specific marker that can be used to identify fibroblasts (Dulbecco et al., 1983; An et al., 2018). Fibroblasts were observed after immunofluorescence staining. Under the microscope, DAPI-stained nuclei emitted blue fluorescence, and vimentin-positive cells emitted red, indicating that the cells obtained were skin fibroblasts (Figures 4C–E); the purity of these fibroblasts was close to 100%.

### Effects of chitooligosaccharides on mitochondrial activity, proliferation and cell cycle of fibroblasts

**FIGURE 5:**
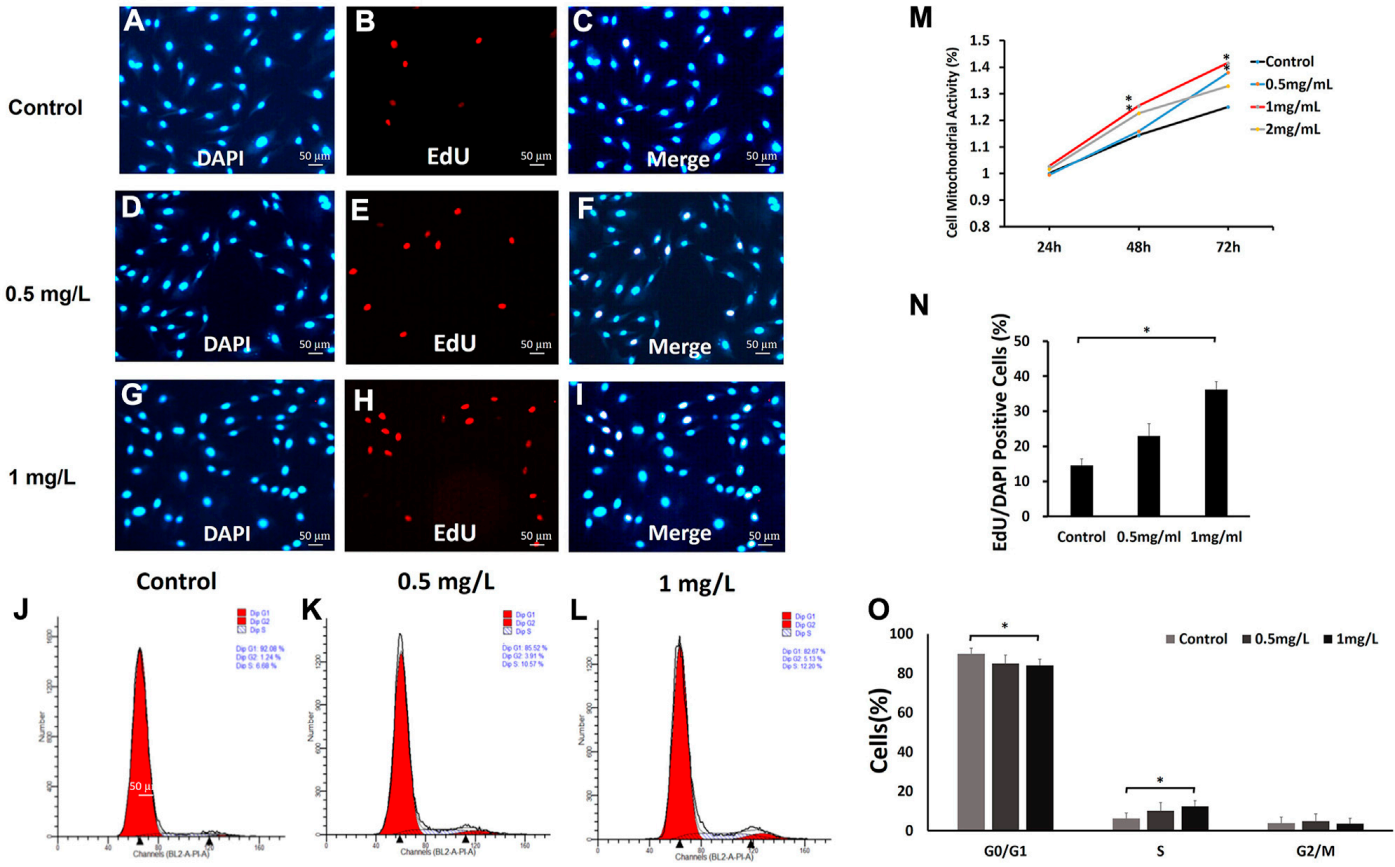
The effects of COS on mitochondrial activity, proliferation and cell cycle of fibroblasts **(A–L,N,O)** The fibroblasts were treated with 0, 0.5, and 1.0 mg/ml COS for 24 and 72 h, and 1.0 mg/ml COS significantly promoted cell proliferation. **(M) **The MTT assays showed that the mitochondrial activity of cells stimulated with COS at 0.5 mg/ml and 1 mg/ml was significantly higher than that of the control group. *p < 0.05. The scale bar represents 50 µm.

Cell mitochondrial activity assays were used to evaluate fibroblast mitochondrial activity after exposure to different concentrations of COS (0, 0.5, 1 and 2 mg/ml) for 24, 48, and 72 h. The results obtained from MTT assays are presented in Figure 5M As shown in Figure 5M, after 24 h, there was no significant difference in cell mitochondrial activity among the four groups. After 48 h, the mitochondrial activity of cells stimulated with COS at 1 and 2 mg/ml was significantly higher than that of the control group. After 72 h, the mitochondrial activity of cells stimulated with COS at 0.5 and 1 mg/ml was significantly higher than that of the control group. Afterward, we used cells with COS concentrations of 0.5 and 1 mg/ml for further experiments.

To further explore the effect of COS on fibroblast proliferation, fibroblasts were treated with 0, 0.5 and 1.0 mg/ml COS for 24 h. We found that 1.0 mg/ml COS could increase cell proliferation by twofold (Figures 5A–I,N). These results indicate that COS facilitates fibroblast proliferation.

The results from the MTT and proliferation assays indicated that a suitable concentration of COS can promote fibroblast mitochondrial activity and proliferation. Then, we examined the effects of 0.5 and 1 mg/ml COS for 72 h on cell cycle distribution by flow cytometry. As shown in Figures 5J–L,O, compared with the control group, the number of cells cultured with 1 mg/ml COS increased significantly in S phase (*p* < 0.05), and decreased in G1 phase (*p* < 0.05), and the number of cells in G2 phase did not change significantly.

### Effect of chitooligosaccharides on cell migration

**FIGURE 6:**
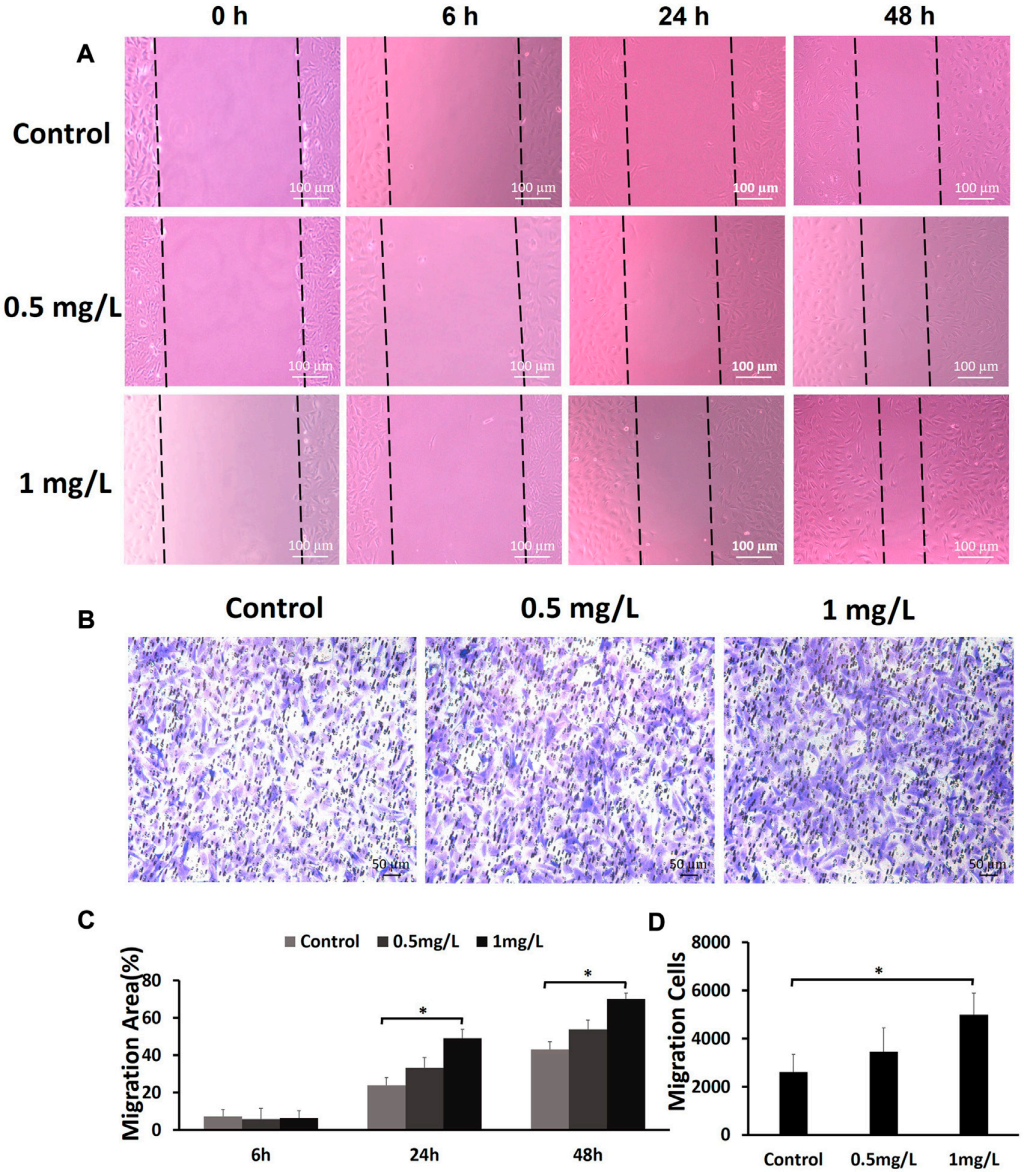
The effects of COS on the migration of fibroblasts **(A)** The wound healing scratch assays showed that 1 mg/ml COS markedly enhanced the motility of fibroblasts. **(B)** The transwell assays showed that compared with the control group, 1 mg/ml COS significantly promoted cell migration. **(C,D)** Quantification of the results from the wound healing assays and transwell assays is shown in the figure. *p < 0.05. The shorter scale bar represents 50 μm, and the longer scale bar represents 100 µm.

To assess the effects of COS on the migration of fibroblasts, cells were subjected to a wound healing scratch assay. The wounded area was photographed at 0, 6, 24, and 48 h after scratching. (Figure 6A). The results showed that 1 mg/ml COS significantly enhanced cell motility, as judged by the wound closure area (Figure 6C). Transwell assays further confirmed the promigratory ability of cells (Figure 6B), which is another method widely used to evaluate cell migration. Transwell assays showed that compared with the control group, the concentration of 1 mg/ml COS significantly promoted cell migration (Figure 6D).

### Functional annotations and expression profiles of genes in chitooligosaccharides-treated fibroblasts

**FIGURE 7:**
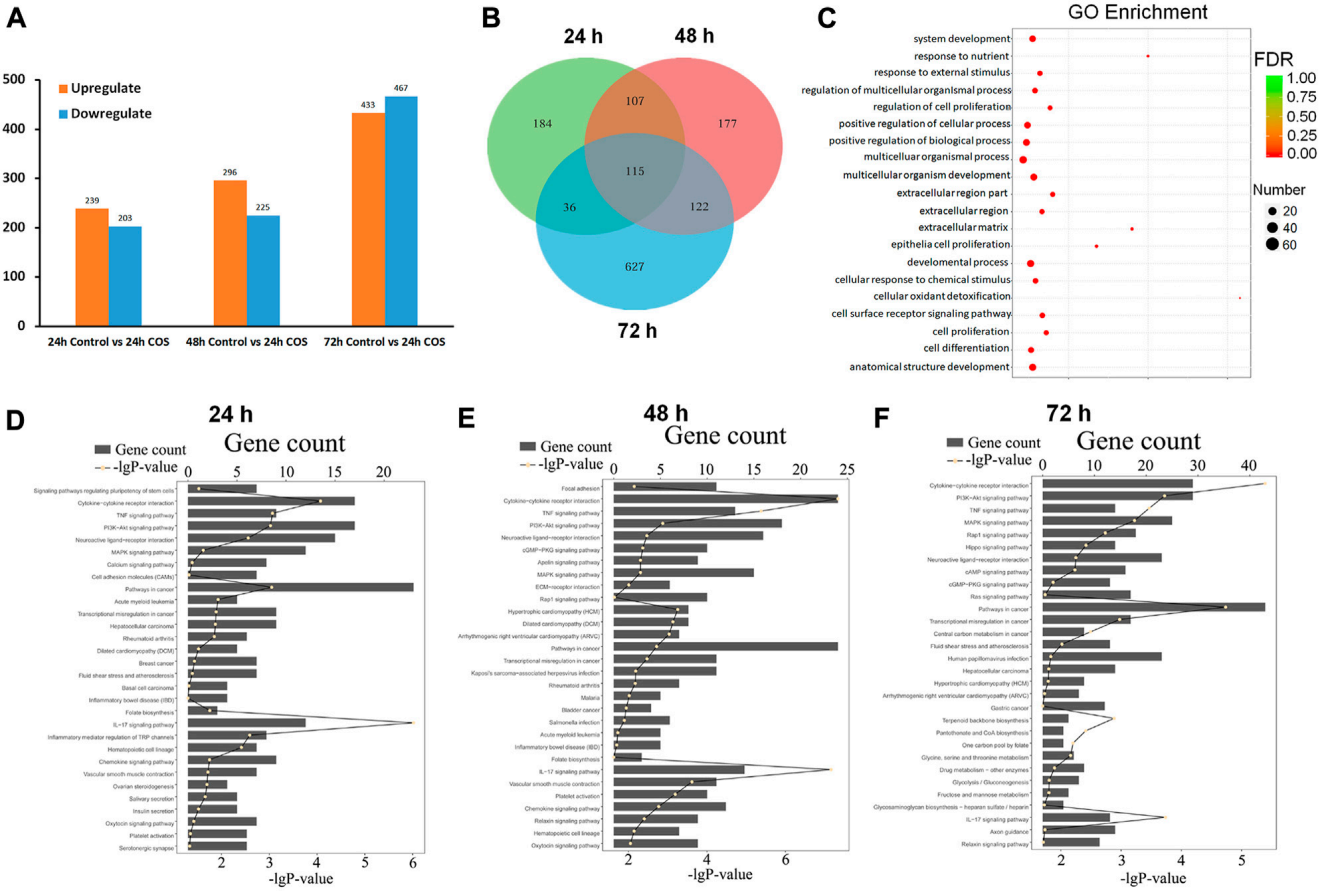
Functional annotations of gens in the COS-treated fibroblasts **(A)** Upregulated and down-regulated genes at each time point. **(B)** The Venn diagram of DEGs **(C)** GO analyses showed 20 significant functional annotations reveals by the target genes. **(D–F)** KEGG analyses showed the most significantly enriched canonical signaling pathways at different time points. The total number of genes is showed in each pathway, and the yellow dots for each pathway indicates −log (*p* value).

We performed transcriptome analysis on fibroblasts treated with or without 1.0 mg/ml COS for 24, 48, and 72 h to compare the genes of cells at three time points. We found 442 DEGs between the control group and the COS treatment group after the cells were cultured for 24 h, (239 of which were upregulated and 203 of which were downregulated), 521 DEGs after the cells were cultured for 48 h, (296 were upregulated and 225 were downregulated), and 900 DEGs after the cells were cultured for 72 h, (433 were upregulated and 467 were downregulated) (Figure 7A). The DEGs were filtered out at the three time points, and a Venn map was drawn. As shown in Figure 7B, there were 115 overlapping genes.

To better understand the function of these DEGs in cells, GO and KEGG annotation analyses were performed from the biological process. Figure 7C shows 20 significant functional annotations related to cell proliferation and differentiation, positive regulation of biological and cellular processes, cellular response to chemical and external stimuli, etc., revealed by the GO analysis of the target genes. KEGG pathway analysis can help to understand the biological processes more systematically and comprehensively, thus, we further analyzed the signaling pathways involved in the DEGs using KEGG. The top 30 enriched KEGG pathways of each comparison are shown in Figures 7D–F, and the top five signaling pathways at the three time points were pathways in cancer, pathways in cytokine-cytokine receptor interaction, PI3K-Akt signaling pathway, mitogen-activated protein kinase (MAPK) signaling pathway, and pathways involved in neuroactive ligand-receptor interaction.

**FIGURE 8:**
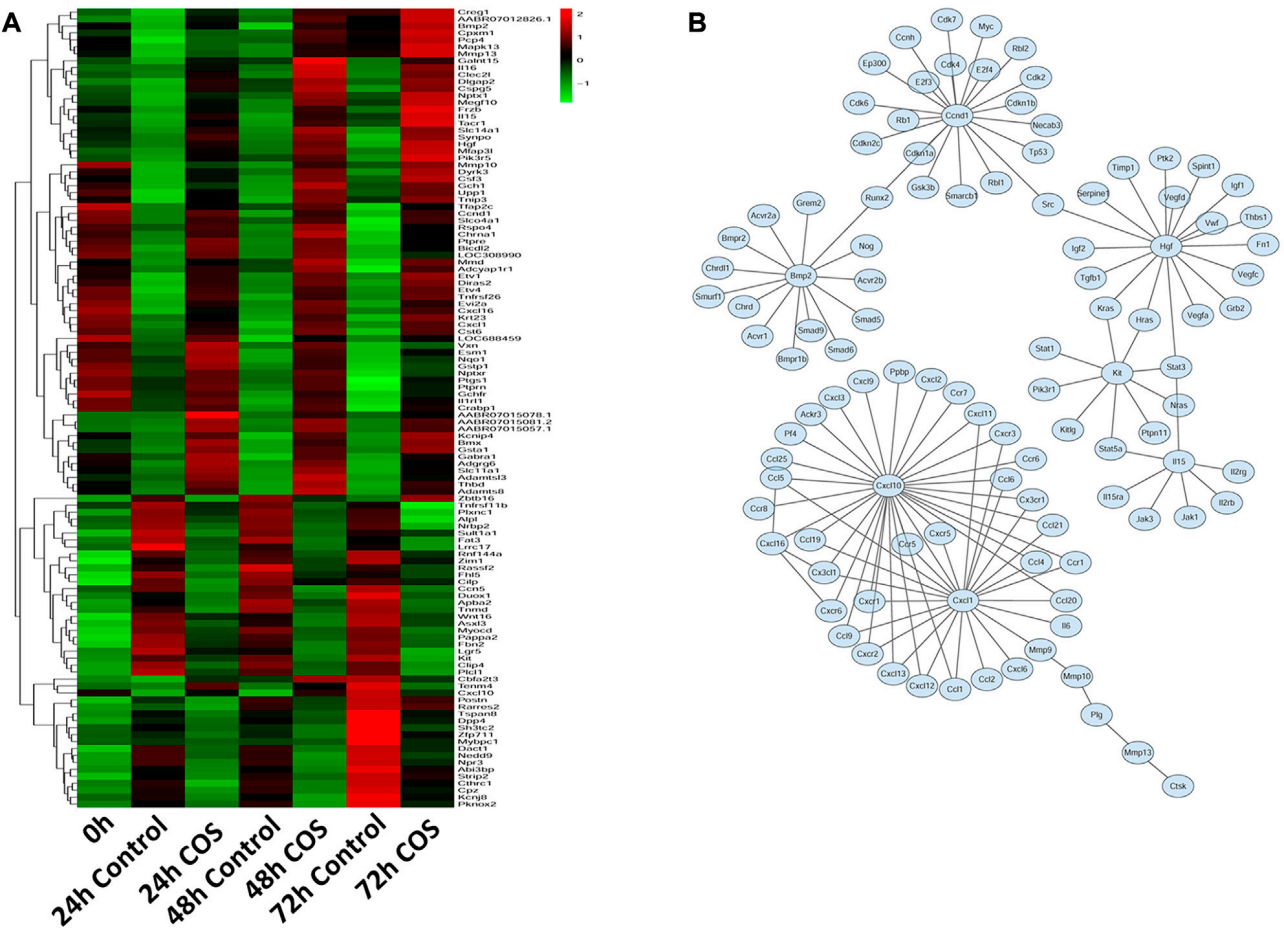
Expression profiles of gens in the COS-treated fibroblasts **(A)** Heatmap and clustering analysis of expression levels of DEGs at each time point. **(B)** Integrated regulatory network of genes inferred by integrating experimental data and computational prediction.

Having determined the genes regulated by COS in fibroblasts, we examined the expression profiles of these genes in response to COS. Hierarchical cluster analysis revealed that multiple genes exhibited dynamic changes between the two groups at 24, 48, and 72 h (Figure 8A). We built a gene network to uncover the most significant pathway targeted by COS (Figure 8B). These results suggest that many genes in cells are regulated by COS. To prove the accuracy of the sequencing results, we selected HGF to verify whether the trends were consistent with the sequencing results.

### The effect of chitooligosaccharides on hepatocyte growth factor expression in fibroblasts

**FIGURE 9:**
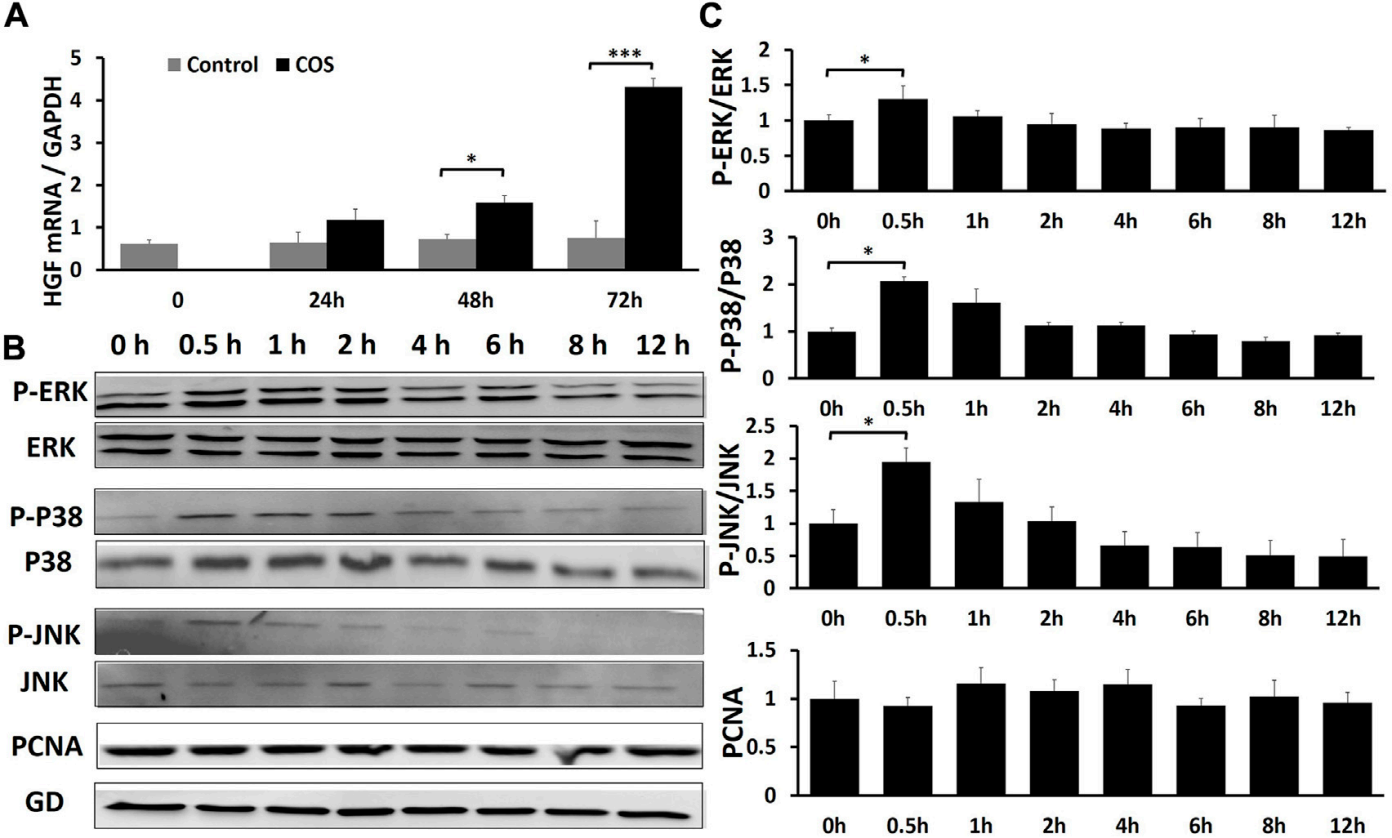
COS can promote wound healing through MAPK pathway and growth factor HGF **(A)** The qRT-PCR indicated that COS may promote wound healing by promoting the expression of HGF. **(B)** Western blotting assays demonstrated that COS stimulated phosphorylation of MAPK proteins (P-ERK1/2, P-p38 and P-JNK) within 0.5 h. **(C)** Quantification of the results from the qRT-PCR and Western blotting assays is shown in the figure. *p < 0.05, ***p < 0.001.

We further investigated the effect of COS on the expression of HGF in fibroblasts. qRT-PCR showed that there was no significant difference in HGF gene expression levels between the two groups at 24 h, but the gene expression levels of HGF were increased significantly at 48 and 72 h in the COS-treated group (*p* < 0.01; Figure 9A). These data showed that COS may promote wound healing by promoting the expression of HGF.

### The effect of chitooligosaccharides on the mitogen-activated protein kinase signaling pathway

Fibroblasts were treated with 1.0 mg/ml COS to observe the effects of COS on the MAPK signaling pathway and cell proliferation within 12 h after stimulation. Briefly, cell lysates were prepared, and the phosphorylation levels of extracellular signal-regulated kinases (ERK1/2), mitogen-activated protein kinase MAPK (p38), c-Jun N-terminal kinase (JNK), and proliferating cellular nuclear antigen (PCNA) in fibroblasts were analyzed by Western blotting. It is generally believed that after stimulation, cells often have an activation peak in the early stage. Similarly, in this experiment, we found that after treatment with 1.0 mg/ml COS, the phosphorylation of ERK1/2, p38 and JNK reached a maximum after 30 min, and then returned to levels close to baseline. And the total ERK1/2, p38 and JNK had no change (Figures 9B,C). It has been demonstrated that COS stimulates a transient and potent phosphorylation of MAPK proteins and their downstream signal proteins. We also found that COS had no significant effect on the level of PCNA protein in fibroblasts within 12 h (Figures 9B,C).

## Discussion

Burns can damage the skin tissue, resulting in the loss of skin barrier function. When the tissue loses the skin barrier, the wound is prone to infection. Deep second-degree burn wounds are common wounds in the clinic. How to effectively promote wound healing and shorten the wound healing time is a hot issue at present. COS is the depolymerization product of chitosan, with various active biological activities such as protecting damaged tissues from infection and supporting cell adhesion, so it has potential therapeutic effects in tissue damage and wound healing (Phil et al., 2018).

As a biodegradable and highly biocompatible biomaterial, COS has attracted researchers in the fields of neurology, orthopedics, and wound repair to conduct extensive and in-depth research on tissue repair. In the field of neurology, studies have shown that COS can improve the mitochondrial activity of Schwann cells, induce their proliferation, and promote the repair and functional recovery of injured nerves. Wang et al. found that COS promoted Schwann cell proliferation by upregulating miR-27a on its target gene FOXO1, thereby promoting nerve regeneration^19^. In the field of orthopedics, studies have shown that COS can treat osteoporosis and promote bone wound healing (Wang et al., 2019). Previous studies have shown that COS can increase femoral strength and bone mineral density and increase the biological activity of bone strength. Ohara et al. showed that COS can upregulate the CD56 gene related to cell differentiation and proliferation at the gene level, which indicates that COS can improve the symptoms of osteoporosis by promoting the proliferation of osteoblasts (Ohara et al., 2004). At present, in the field of wound repair, studies have shown that COS can accelerate the secretion of type III collagen, promote epithelium and granulation tissue formation, and promote wound healing (Park et al., 2018). The role of COS in the field of tissue repair is multifaceted.

In this study, we used COS to treat deep second-degree burn wounds in rats. Rats in the scald COS treatment group were injected with 0.2 ml (0.2 mg/ml) of COS solution around the wound every other day for a week and a scald control group was established to evaluate the therapeutic effects of COS. COS can promote deep burn wound healing effectively (Figures 2E,F,O). All rats were fully healed 3 weeks after injury, and the complete epithelialization rate of the COS-treated group was significantly higher (Figures 2G,H,P). H&E staining proved that the skin tissue appendages of COS-treated rats were also significantly increased after healing (Figures 2Q–X). Compared with the rats in the sham scald group, the tissue water content showed that 2 days after injury, the wound tissue of scald rats showed obvious edema (Figure3Z). The wound blood flow of rats in the two scald groups was detected with a blood perfusion measuring instrument. We found that the blood flow of the rats treated with COS was significantly higher than that of the control group 3 weeks after injury (Figures 3A–Y).

Deep second-degree burns not only destroy epidermal cells but also damage many dermal cells in skin tissue (Kagan et al., 2013). Wound repair involves a series of stages, which include homeostasis, inflammation, proliferation, and remodeling (Rowan et al., 2015; Wang et al., 2018). In the biological process of deep burn wound repair, fibroblasts promote wound healing through migration, proliferation, and cell differentiation (Zuliani et al., 2013; Pezeshki-Modaress et al., 2014; Jiang et al., 2020). At present, many studies have confirmed that fibroblasts are important cells in wound healing (Tettamanti et al., 2004; Haghshenas et al., 2019). Therefore, we cultured fibroblasts from the back skin of young SD rats (3 days old), and the obtained cells were identified by vimentin immunofluorescence and confirmed to be fibroblasts. Then, we used COS to stimulate the fibroblasts. We found that 1 mg/ml COS not only promoted the mitochondrial activity and proliferation of fibroblasts but also significantly promoted cell migration and cell motility.

Recently, the development of RNA-seq has greatly expanded our understanding of biological systems, and gene expression profiling using RNA-seq is currently a core activity in molecular biology (Chisanga et al., 2022). Our *in vivo* experimental study found that COS can effectively promote the healing of deep burn wounds in rats. The *in vitro* experiment showed that COS can effectively promote the proliferation and migration of fibroblasts. Revealing the molecular mechanism by which COS promotes wound healing and fibroblast proliferation is helpful to guide clinical practice. We used RNA-seq to examine the gene expression profile of COS-treated fibroblasts and found 442, 521 and 900 DEGs between the two groups at the three time points (Figure 7A). After skin damage, various cells are activated under endogenous or exogenous stimulation; for instance, cell proliferation, cell division and ECM remodeling will occur, creating conditions for wound healing (Li, et al., 2007). Analysis of variable gene-related signaling pathways by GO and KEGG identified that cell proliferation and differentiation, cellular response to chemical and external stimuli, cytokine-cytokine receptor interaction, PI3K-Akt signaling pathway, MAPK signaling pathway and other signaling pathways were changed (Figures 7C–F). We also constructed a network (Figure 8B) and found that many genes in cells are regulated by COS. We studied and analyzed genes associated with cell proliferation, migration, and MAKP signaling pathways at three time points. As shown in Supplementary Figure S1, the genes related to proliferation that co-upregulated at three time points were: ETV4, DYRK3, CHRNA1, CREG1, BMP2, MMD; the genes related to migration that co-upregulated at three time points were: TACR1, ESM1, MMP13, ETV1, GSTP1, PIK3R5, CXCL1, CXCL16, IL1, RL1, PTGS1, CST6, BMP2; and genes related to MAPK signaling pathway that co-upregulated at three time points were: ETV4, MMP10, HGF. HGF is a ubiquitous and multifunctional cytokine that can be expressed and secreted in normal human cells, and it is essentially a polypeptide growth factor (Randhawa et al., 2013). HGF has been shown to stimulate proliferation of various types of cells and have multiple activities such as motogenic, morphogenic and mitogenic activity (Zarnegar and Michalopoulos, 1995; Matsumoto and Nakamura, 1997). We further studied the effect of COS on the expression of HGF in fibroblasts to verify whether the trends were consistent with the sequencing results. The data demonstrated that COS stimulated the expression of HGF (Figure 9A). These results show that the effect of COS on fibroblasts involves multiple genes and related signaling pathways. It may be owing to the effect of COS that some signaling pathways are altered to promote cell proliferation and migration, thereby promoting the healing of burn wounds in rats.

The MAPK family is widely distributed in mammals. It is a group of major signaling molecules in the process of signal transduction and plays an important role in the development of diseases. The MAPK family mainly includes JNK, p38, ERK1/2, and other subfamilies. These subfamilies are the hubs of the signaling pathway and play an important role in modulating various cellular functions (Colecchia, et al., 2012; Lanna, et al., 2017). The MAPK signaling pathway regulates various physiological and pathological processes in cells and is closely related to cell proliferation, migration, differentiation, maintenance of cell morphology, cell damage and repair. Extracellular signals such as inflammatory cytokines, growth factors or some physical stimuli can activate MAPKs. When activated, the protein phosphorylation level increases, which mediates a series of cell proliferation, differentiation, migration and inflammatory responses (Wang, et al., 2003; Huang, et al., 2004; Hatzivassiliou et al., 2010). Then we observed the effects of COS on the MAPK signaling pathway. We found that the protein levels of phosphorylated JNK, p38 and ERK1/2 (P-JNK, P-p38 and P-ERK1/2) reached a maximum after stimulated with COS for 30 min (Figures 9B,C). These results indicated that COS may promote the proliferation and migration of fibroblasts by stimulating the MAPK signaling pathway.

There are few studies on the effects of COS on fibroblast signaling pathways. In MAPK signaling pathway, p38 is one of the key factors in the regulation of cell proliferation and differentiation (Li et al., 2021). We chose the p38 pathway to further verify that COS could promote wound healing through MAPK signaling pathway. The fibroblasts were pretreated with 20 μM SB203580 (p38 inhibitor, Med Chem Express) for 1 h and stimulated with 1 mg/ml COS, then they were compared with the cells that were normal cultured and the cells cultured with 1 mg/ml COS. As shown in Supplementary Figures S2–S5, 1 mg/ml COS can significantly increase the mitochondrial activity, proliferation and migration of fibroblasts as compared with the control group. The p38 inhibitor, SB202190 (20 μM) completely abrogated the activation effect of COS on the fibroblasts. Thus, the enhancement of mitochondrial activity, proliferation and migration of fibroblasts may be dependent on the early activation of p38.

It has been reported that p38 overexpression stimulates the production of matrix by dermal fibroblasts, leading to the hyperplasia of scars (Bhowmick et al., 2001; Li et al., 2001; Tsukada et al., 2005). It has also been suggested that p38 overexpression may be associated with fibrosis-related processes observed in the heart (Turner and Blythe, 2019), lungs (Chen et al., 2013), liver (Tang et al., 2013), and kidneys (Sugiyama et al., 2012). We found that the wound healing time of COS-treated rats was shorter, and there was no obvious scar hyperplasia (Figures 2I–M,J–N). Similarly, taking p38 signaling pathway as an example, *in vivo* experiments, immunohistochemical staining on tissue of COS-treated rats showed lower expression of p38 compared to the rats in the scald control group 1 week after injury (Supplementary Figure S6B–6C1). Therefore, stimulation with COS on the phosphorylation of p38 in fibroblasts is transient and effective. COS temporarily activated fibroblasts through p38 signaling pathway in the early stage, promoting their proliferation and migration to promote wound healing, and will not lead to scar hyperplasia.

## Conclusion

This study showed that COS can not only promote the healing of wounds but also promote the proliferation of fibroblasts. We performed transcriptome analysis on fibroblasts treated with COS and found a large number of DEGs between the control group and the COS treatment group. GO and KEGG annotation analyses were performed from the biological process category, and we found that GO analysis of the target genes revealed 20 significant functional annotations and that the target genes of KEGG analysis revealed 30 enriched KEGG pathways. The key sequencing results were verified by qRT-PCR and Western blotting assays, and we preliminarily proved that COS can promote wound healing through the MAPK pathway and growth factor HGF. The mechanism by which COS promotes wound healing remains to be further explored and studied.

## Data Availability

The datasets presented in this study can be found in online repositories. The names of the repository/repositories and accession number(s) can be found below: https://www.ncbi.nlm.nih.gov/, PRJNA860830.
