# Supplementary material for: Chitosan degradation products promote healing of burn wounds of rat skin
Source: Front Bioeng Biotechnol. 2022 Oct 11;10:1002437. doi: 10.3389/fbioe.2022.1002437 (PMC9592717; doi:10.3389/fbioe.2022.1002437)
Supplement: Supplementary file 1 [file DataSheet1.docx]

**Supporting Information**

Chuwei Zhang‡1, Qingrong Zhang‡2,3, Dongmei Yang‡^4^, Yating Qiao^5^, Bolin Wang1, Jun Yan1, Zihan Li^1^, Zhanghao Huang^6^, Youlang Zhou ^*7^, Kesu Hu *2, Yi Zhang *2

^1^Department of Burn and Plastic Surgery, Affiliated Hospital of Nantong University, Medical School of Nantong University, Nantong, 226001, China

^2^Department of Burn and Plastic Surgery, Affiliated Hospital of Nantong University, Nantong 226001, China

^3^Third Military Medical University (Army Medical University), Chongqing, 400038, China.

^4^Outpatient treatment center, Department of Burn and Plastic Surgery, Affiliated Hospital of Nantong University, Nantong 226001, China

^5^Affiliated Hospital of Hebei University, Baoding, 071000, China

^6^Department of Thoracic Surgery, Affiliated Hospital of Nantong University, Medical School of Nantong University, Nantong 226001, China

^7^The Hand Surgery Research Center, Department of Hand Surgery, Affiliated Hospital of Nantong University, Nantong, 226001, China

*Corresponding authors:

Youlang Zhou, Email: youlangzhou@163.com;

Kesu Hu, Email: [gc-sh@163.com](mailto:gc-sh@163.com);

Yi Zhang, Email: [198zy@163.com](mailto:198zy@163.com).

**‡These authors contributed equally to this work**

1. **Materials and methods**

We studied and analyzed genes associated with cell proliferation, migration, and MAKP signaling pathways at three time points. Deviation map is designed to filter the differential genes whose P value is greater than or equal to 0.05 and Log2 | Foldchange | > = 1.

**1.2** **Cell Mitochondrial Activity Assay**

Cell Mitochondrial Activity was assessed by MTT [3-(4, 5-dimethylthiazol-2-yl)-2, 5-diphenyltetrazolium bromide] assays. Fibroblasts were seeded (200 µL, 1 × 10^4^ cells / mL) in 96‑well plates for 24 h. Then the cells were preincubated for 1 h with 20 μM SB203580 (p38 inhibitor, Med Chem Express) before treatment with 1 mg/mL COS for 48 h and finally incubated with 20 µL of MTT (5 mg/mL, Sigma) for 4 h. Then removed the supernatant, and added 150 µL of dimethyl sulfoxide (DMSO), after shaking the plates in the dark for 10 min, determined the absorbance D (570) value of cells at 570 nm on a microplate reader (ThermoFisher, USA). Untreated cells with 100% mitochondrial activity were used as control, and the experiment was repeated three times.

**1.3 Fibroblasts** **Proliferation Assay**

The fibroblasts were seeded onto 0.01 % poly-L-lysine-coated 96-well plates with an initial density of 1 × 10^5^ cells/well in 0.2 mL of culture medium and incubated at 37 ℃ and 5% CO_2_ for 24 h. Then the cells were preincubated for 1 h with 20 μM SB203580 (p38 inhibitor, Med Chem Express) before treatment with 1 mg/mL COS for 24 h. Finally, fixed the cells with 4 % formaldehyde for 30 min in PBS. According to the manufacturer’s protocol, using a Cell Light EdU DNA Cell Proliferation Kit (Ribobio) to assay the fibroblasts after labeling. Under a fluorescence microscope, the proliferation of fibroblasts (ratio of EdU+ to all fibroblasts) was analyzed with randomly selected fields images. All experiments had three replicates.

**1.4** **The wound healing assay**

The wound healing assay was performed to study the effect of COS on the migratory capacity of fibroblasts. Seeded the fibroblasts (3 mL/well, 1 × 10^6^ cells / mL) into 6-well plates and incubated at 5% CO_2_ and 37 ℃. After the cells reached 80–90% confluence, subjected the cells to starvation for 24 h in low serum (1% FBS) medium. Scratched the monolayer with a sterile 1000 μL pipette tip and removed the exfoliated cells with PBS. Then the cells were preincubated for 1 h with 20 μM SB203580 (p38 inhibitor, Med Chem Express) before treatment with 1 mg/mL COS, at 0 h, 24h, 48 h, and 72 h postwounding, scratched fields were photographed, and wound closure area was calculated as follows: Migration area = (wound area on initial - the remaining area of wound at each time point)/(wound area on initial) × 100%.

**1.5 Migration Assay**

The transwell assay was performed using the cell culture insert (FALCON, NJ, USA).

The 24-well plate was added with complete medium supplemented with1 mg/mL COS as the lower chamber. The cell culture insert (FALCON, NJ, USA) was placed into each well, and the top was pressed lightly to ensure tight bonding (Zhao et al., 2017). The normal cultured fibroblasts and the SB203580 pre-treated fibroblasts (4 × 10^3^ cells/well) were suspended in low serum medium and seeded into the cell culture insert (FALCON, NJ, USA) with an 8 μm pore size as the upper chamber. After culturing for 24 h, the cells attached to the upper surface of the filter membranes were removed, and stained the migrated cells on the lower surface for 10 min with 0.5% crystal violet. The migration level was observed under a microscope.

**1.6 Immunohistochemical Staining Analysis**

Immunohistochemistry was used to detect the expression of p38 and in the wound of rats one week after injury, and the normal skin was taken as control. The slides were incubated with 3% hydrogen peroxide at room temperature for 5~10 min to block endogenous peroxidase activity. The water bath was set to 100 ℃, and the slides were placed in citrate buffer (Dako, Glostrup, Denmark) solution for 5 min for antigen repair. Next, the slides were incubated with primary antibodies at 4 ℃ overnight and rinsed with PBS, blocked with 5% BSA at 4 ℃ overnight. Then incubated the slides with a rabbit anti-p38 antibody (1:1000, GB11005, Servicebio) at 4 ℃ overnight. After that, washed the slides with PBS and incubated with secondary antibody at 4 ℃ overnight. Stained the slides with 3-3′-diaminobenzidine (DAB, Sigma, St. Louis, MO, USA) for a color development and then counterstained with hematoxylin. Then sealed the slides with neutral gum (Invitrogen, San Diego, CA, USA), and a microscope (Leica DMR 3000, Leica Microsystems, Bensheim, Germany) was used to observe and evaluate tissue images.

**2. Results**

**
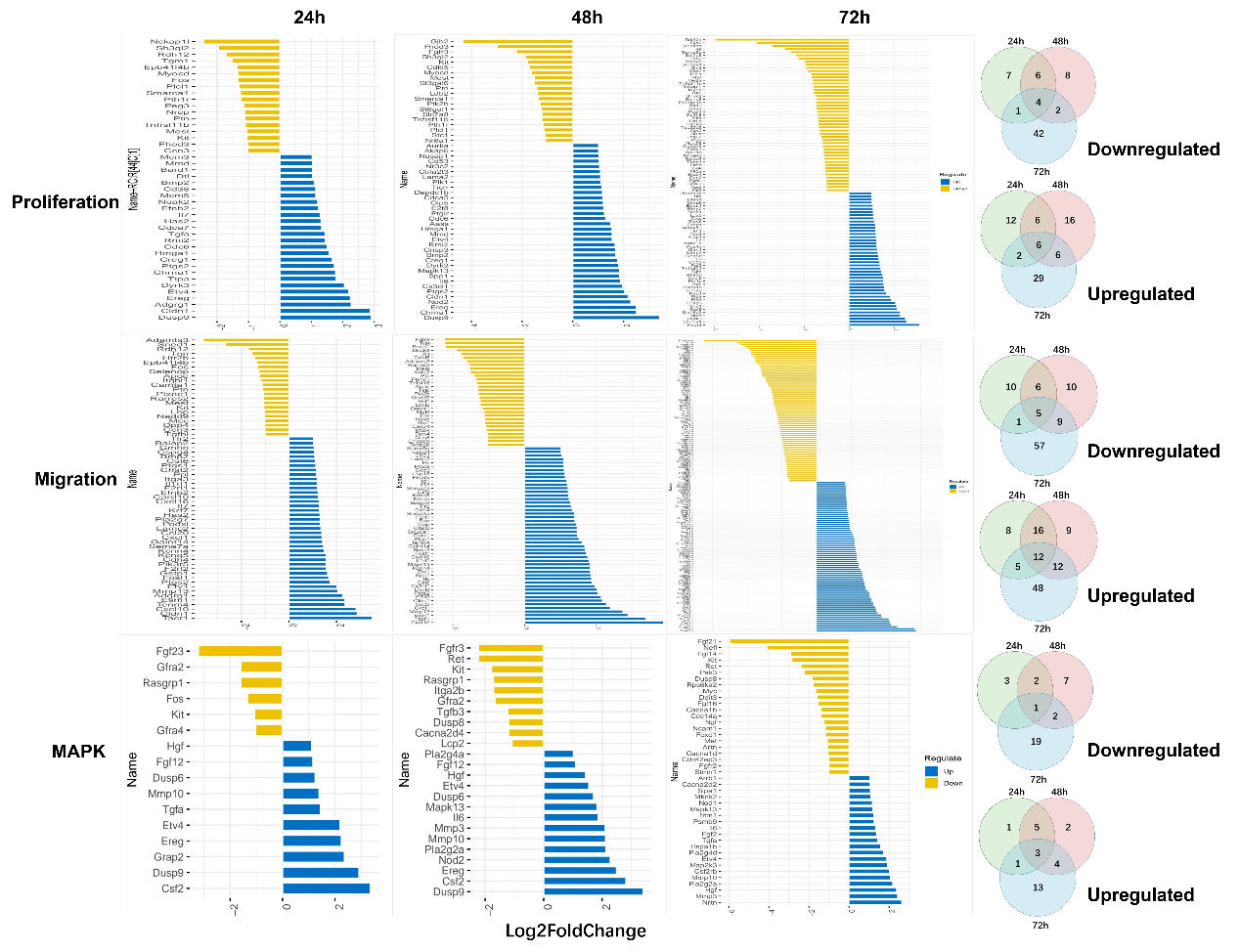
**

**FIGURE S1** | Genes associated with cell proliferation, migration, and MAKP signaling pathways at three time points.

**
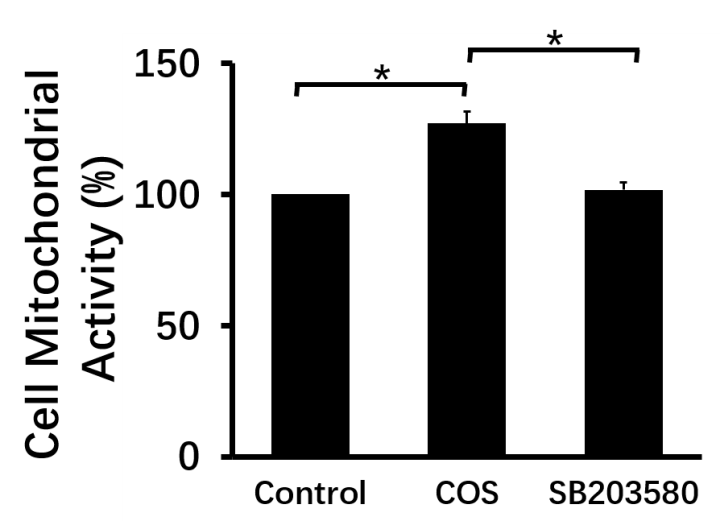
**

**FIGURE S2** | The effects of SB203580 and COS on mitochondrial activity of fibroblasts. The MTT assay showed that 1 mg/mL COS can significantly increase the mitochondrial activity of fibroblasts as compared with the control group, and SB202190 (20 μM) completely inhibited the effect of COS on the fibroblasts. * P < 0.05.

**
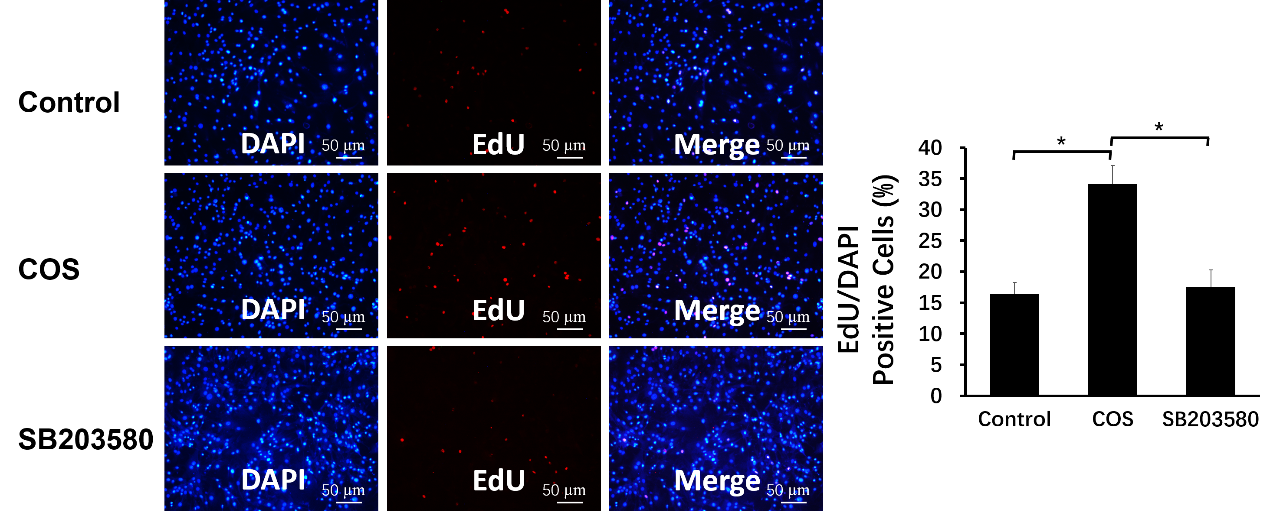
**

**FIGURE S3** | The effects of SB203580 and COS on proliferation of fibroblasts. The proliferation assay showed that 1 mg/mL COS can significantly increase the proliferation of fibroblasts as compared with the control group, and SB202190 (20 μM) completely inhibited the effect of COS on the fibroblasts. * P < 0.05. The scale bar represents 50 µm.

**
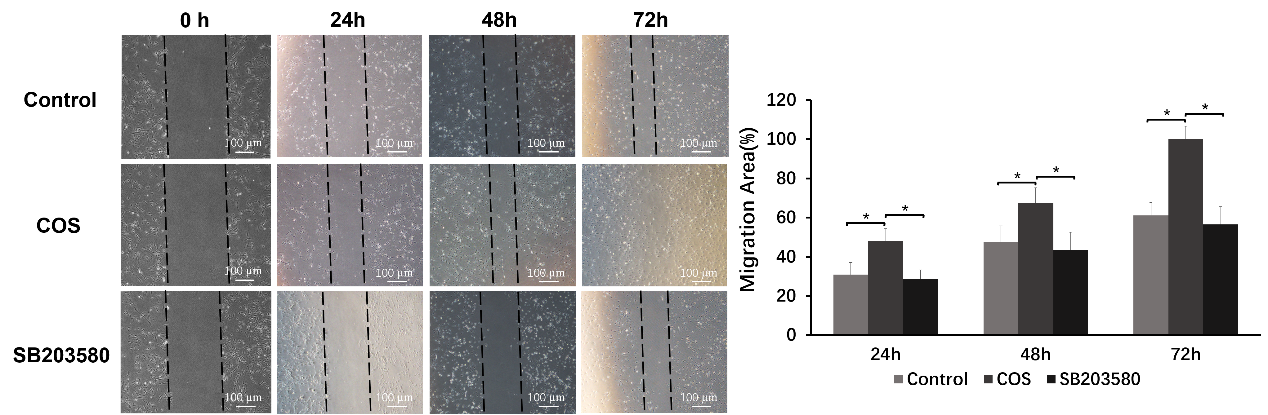
**

**FIGURE S4** | The effects of SB203580 and COS on migration of fibroblasts. The migration assay showed that 1 mg/mL COS can significantly promote the migration of fibroblasts as compared with the control group, and SB202190 (20 μM) completely inhibited the effect of COS on the fibroblasts. * P < 0.05. The scale bar represents 100 µm.


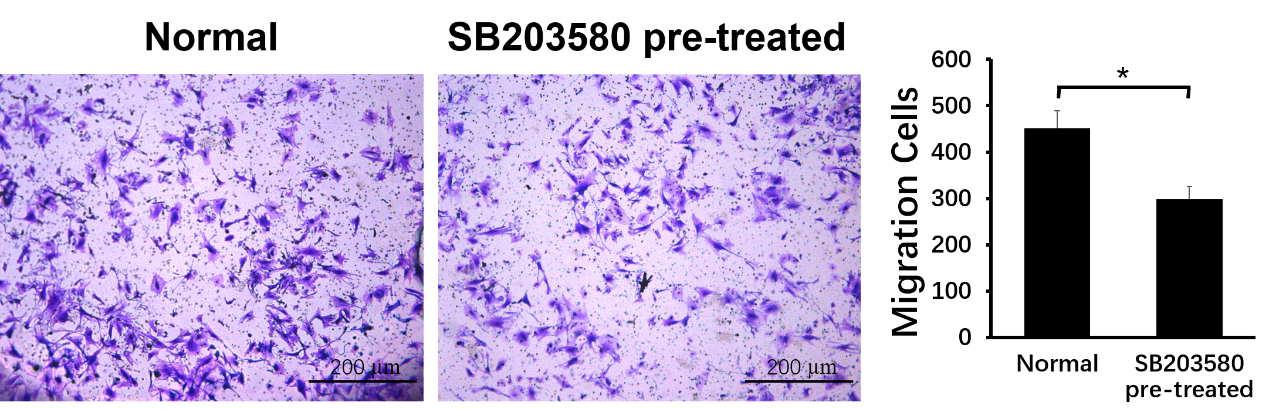


**FIGURE S5** | The effects of SB203580 on the migration of COS-treated fibroblasts. (A-B) The transwell assay showed that compared with the normal cultured fibroblasts, the number of SB203580 pre-treated fibroblasts was significantly reduced. (C) Quantification of results from the transwell assay in figure. * P < 0.05. The scale bar represents 50 µm.

**
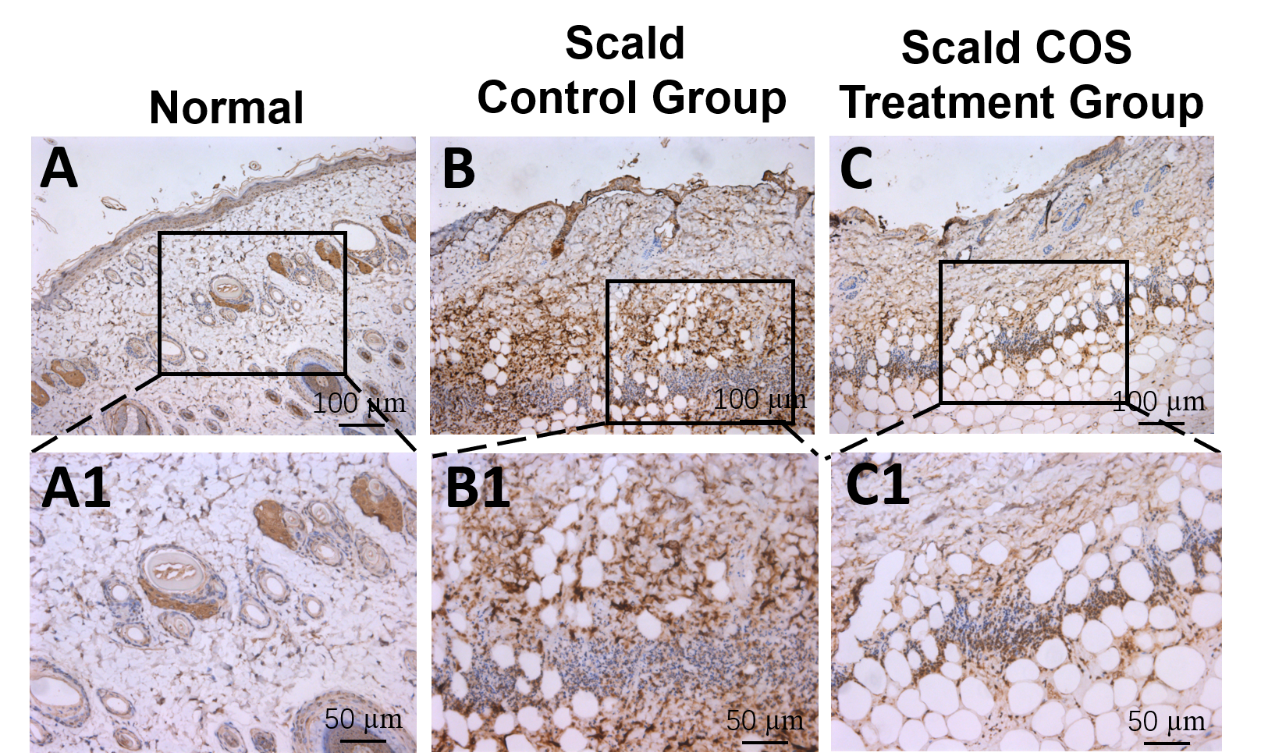
**

**FIGURE S6** | Immunohistochemical staining of p38 in the tissues one week after injury. A few p38 positive cells were found in normal skin tissues of rats, which were mainly distributed in hair follicles, sebaceous glands and epidermis. Immunohistochemical staining on tissue of COS-treated rats showed lower expression of p38compared to the rats in the scald control group. Each right photo showing a histological section is a magnification of the rectangle-delimited area in the corresponding left photo. The shorter scale bar represents 50 µm, and the longer scale bar represents 100µm.
